# Clinical Characteristics, Management, and Outcomes of 19 Nonpediatric Patients with Desmoplastic Small Round Cell Tumor: A Cohort of Brazilian Patients

**DOI:** 10.1155/2020/8713165

**Published:** 2020-10-28

**Authors:** Fernando Campos, Daniel L. Coutinho, Maria Letícia G. Silva, Ademar Lopes, Antônio Nascimento, Samuel Aguiar Júnior, Ulisses R. Nicolau, Maria Nirvana Formiga, Felipe D'Almeida Costa, Celso Mello

**Affiliations:** ^1^Department of Medical Oncology, A. C. Camargo Cancer Center, São Paulo, SP, Brazil; ^2^Department of Radiation Oncology, A. C. Camargo Cancer Center, São Paulo, SP, Brazil; ^3^Department of Surgical Oncology, A. C. Camargo Cancer Center, São Paulo, SP, Brazil; ^4^Department of Pathology, A. C. Camargo Cancer Center, São Paulo, SP, Brazil

## Abstract

Desmoplastic small round cell tumor (DSRCT) is a rare and aggressive mesenchymal malignancy, usually affecting young males. There is no consensus on the best therapeutic approach. We seek to characterize a cohort of nonpediatric patients with DSRCT treated at a large Brazilian cancer center. We performed a retrospective analysis of patients with histologically confirmed DSRCT referred to our institution (2007–2020). Clinical and imaging data were extracted and summarized with descriptive statistics. Survival analyses were conducted by the Kaplan–Meier method and compared with the log-rank test. We included 19 patients with DSRCT, the median age at diagnosis was 26 years (range: 15–41 years), and 68% were male. Ninety percent presented with abdominopelvic masses, and 32% had extra-abdominal metastasis at diagnosis. Eleven patients (58%) underwent surgery, four patients (21%) received whole abdominal adjuvant radiotherapy, and five patients (26%) had hyperthermic intraperitoneal chemotherapy. Median OS was 27 months (interquartile range: 18–51 m). The five-year OS rate was 12%. Our data confirm the aggressiveness of DSRCT despite intense multimodality treatment. Outcomes of patients treated in a reference cancer center in a developing country are similar to cancer centers in developed nations. Multicenter cooperation is urgent to the development of clinical trials and to improve diagnosis and treatment efficacy.

## 1. Introduction

Desmoplastic small round cell tumor (DSRCT) is a very rare type of soft tissue sarcoma. It is estimated an incidence between 0.2 and 0.5 new cases per million per year [1]. The disease was first described as a new entity in 1989 by Gerald and Rosai in an 8-year-old girl presenting a 16 cm intra-abdominal mass [2]. They proposed this tumor would develop from a pluripotent cell capable of multi-immunophenotypic differentiation. DSRCT has a peak of incidence in adolescents and young adults, with a median age of approximately 19–22 years, ranging from 1.5–74 years [3–5]. There is strong male predominance, and the male-to-female ratio is 4:1 [6]. It predominantly originates from the peritoneum or retroperitoneum and can invade the omentum with multiple peritoneal implants involving the diaphragm, splenic hilum, mesentery of the small and large bowel, and the pelvic peritoneum [4,7,8]. Other sites of the primary tumor are also described in the literature [9,10].

Cytogenetic and molecular characterization of DSRCT has identified a unique chromosomal rearrangement, *t*(11; 22) (*p*13; *q*12), associated with this tumor [11,12]. Furthermore, emerging evidence of genome-wide studies suggests other potential genes to be associated with tumorigenesis [13]. There is no consensus on the best therapeutic approach, although multimodal therapy combining multiagent intensive chemotherapy and aggressive debulking surgery, with a role for adjuvant radiotherapy, appears to represent the standard of care for patients presenting without extra-abdominal metastases [14].

Herein, we present a series of 19 adult and adolescent patients with DSRCT treated at our cancer center aiming to analyze treatment and outcome patterns, searching for prognostic information.

## 2. Materials and Methods

### 2.1. Patients

This study was conducted after approval from the ethics committee. Medical records of patients who received treatment for DSRCT at A. C. Camargo Cancer Center (ACCCC) between January 1, 2007, and January 30, 2020, were reviewed, and pathologic diagnosis review was performed if the material was available. The minimum age at diagnosis to be enrolled was 14 years. DSRCT diagnosis was established by histology and immunohistochemistry, and/or cytogenetics by a sarcoma-specialized pathologist at ACCCC. If biopsy was performed outside our institution, it was reviewed by a sarcoma pathologist at ACCCC. Cases referred to our hospital exclusively for pathology consultation were excluded. The extracted data included demographic characteristics, clinical presentation, radiological findings, histopathology characteristics, treatment patterns, and follow-up information. Data were gathered from electronic clinical charts, and a retrospective database was constructed for analysis.

### 2.2. End Point Definition

Date of diagnosis was defined as the date of biopsy-proven disease. Overall survival (OS) was calculated from the date of the diagnosis to the date of death or the end of the follow-up period (April 28, 2020). Progression-free survival (PFS) was considered from the date of the beginning of treatment to the date of disease progression or death by any cause. Completeness of cytoreduction scores (CCR) was defined as follows: CC0 as complete resection to microscopic disease, CC1 as <2.5 cm^2^ gross residual tumor after resection, and CC2 as gross residual disease >2.5 cm^2^. Radiological disease progression was defined according to Response Evaluation Criteria in Solid Tumors (RECIST). All patients were retrospectively staged according to the MD Anderson Cancer Center DSRCT staging criteria [15].

### 2.3. Statistical Analysis

Statistical analyses were performed on SPSS version 23. Data were expressed as median with the interquartile range (IQR) and percentage unless otherwise stated. Patients who did not die during the study period were censored at their most recent follow-up at ACCCC. Survival analyses were conducted by the Kaplan–Meier method and compared with the log-rank test. The Cox regression model was used to estimate the hazard ratio (HR) of overall survival (OS). HRs are presented with a 95% confidence interval (95% CI), and statistical significance was defined as *p* value <0.05.

## 3. Results

### 3.1. Patient Characteristics

Twenty-five patients were identified at our institutional database with an initial diagnosis of DSRCT. Six patients were excluded: three of them had their tumor reclassified as another sarcoma other than DSRCT by our sarcoma pathologists, two patients had just one appointment for a second opinion with our sarcoma team, and one patient had the first diagnosis at our center but was treated at an external site.

The study included 19 patients, 13 males (68%) and 6 females (32%). The mean age at diagnosis was 26 years (range: 15–41 years).  Table 1 summarizes the characteristics of the patient cohort. All patients presented symptoms at diagnosis, the most common being abdominal pain (56%), weight loss (35%), and ascites (33%). Five patients were initially treated with chemotherapy in other institutions before being referred to our center.

Six patients (32%) had a family history of cancer, none of them with a pattern of some hereditary cancer syndrome that required further genetic investigation.

### 3.2. Radiological Findings and Diagnosis

Computer tomography images of abdomen, pelvis, and chest were the preferred method for the initial evaluation of disease spread. The most common radiologic finding was solid masses with necrotic and liquefaction areas in the center within the peritoneal cavity. In two patients, areas of calcification inside the tumors were also described. The main location of the tumors was in the peritoneum, but two patients had a single well-defined mass located in the thoracic cavity, without abdominal involvement. The median largest dimension of the primary tumor was 13 cm (IQR: 16–11 cm). The majority of patients (79%) presented lymphadenopathy, which was located in abdomen (*n* = 11), thorax (*n* = 2), or both cavities (*n* = 2). Ten patients (53%) had visceral metastasis at diagnosis, and the main site was liver (*n* = 8). One patient had breast metastasis and another two patients presented bone metastasis.

Eleven patients (58%) had the first diagnosis of malignancy at another institution before starting treatment at our center. Of those, 4 patients (36%) received a different diagnosis from DSRCT before review by one of our sarcoma pathologists, one of which was treated with 4 lines of chemotherapy (cisplatin/etoposide, irinotecan, carboplatin/paclitaxel, and gemcitabine/oxaliplatin) without response to any of them before admission in our institution.

### 3.3. Treatment

The treatments are summarized in Table 2. For all patients, a multidisciplinary team (MDT) discussion evaluation was proposed before starting treatment at our center. Eleven patients (58%) were submitted to cytoreductive surgery (CRS) and it was considered complete (CC0) in 10 (53%). Five of those patients also received hyperthermic intraperitoneal chemotherapy (HIPEC). In 4 patients, HIPEC was performed with doxorubicin and cisplatin, and in the other one, doxorubicin and docetaxel were used. Four patients were submitted to whole abdominopelvic radiation therapy (WAP-RT) with a total dose of 30 Gy. Another patient had completed 3 sessions of WAP-RT until being diagnosed with progressive disease in the abdomen. Radiation was interrupted and palliative chemotherapy initiated. All patients received chemotherapy with a median of 2.5 lines (range: 1–7). The main chemotherapeutic drugs utilized in combination were doxorubicin, cyclophosphamide, vincristine, ifosfamide, and etoposide. In the first line, the combination of vincristine/doxorubicin/cyclophosphamide (VAC) intercalated with ifosfamide/etoposide (IE) was used in 37% of patients (*n* = 7), followed by VAC (16%, *n* = 3) and VAI (16%, *n* = 3). Nine patients (47%) received neoadjuvant chemotherapy. Of those, 7 patients (78%) presented a partial response, 1 patient (11%) had stable disease, and 1 (11%) presented progressive disease. One patient who was referred to our center for cytoreductive surgery received neoadjuvant chemotherapy in the original institution with carboplatin, paclitaxel, and bevacizumab.

Out of 11 patients who underwent surgery, one patient went through a new CRS for recurrent disease 10 months after the first surgery, but it was a CC2 due to unresectable liver metastatic lesions.

### 3.4. Follow-Up and Survival

**Figure 1:**
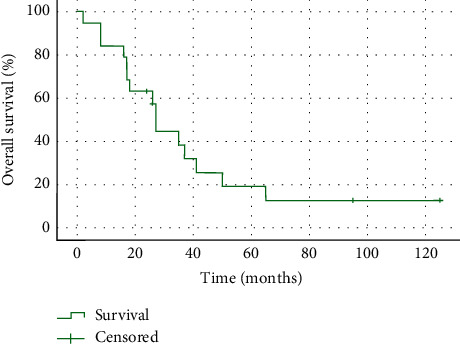
Overall survival for the entire cohort (*n* = 19).

After a median follow-up of 95 months (range: 125 m–95 m), the median OS for the entire cohort (Figure 1) was 27 months (IQR:18 m–51 m). One-, three-, and five-year OS rates after diagnosis were 84%, 38%, and 12%, respectively. One patient died of postoperative complications after receiving neoadjuvant chemotherapy and being submitted to a CC0 surgery without HIPEC. All other deaths were cancer-related. At the end of the follow-up period, 5 patients were alive, 2 of them with active disease and 3 patients in complete remission. Two of the surviving patients had abdominopelvic DSRCT, one presenting a 12 cm single intra-abdominal lesion and the other with multiple peritoneal masses. Both received neoadjuvant chemotherapy, the former VAC/IE protocol and the latter 6 cycles of doxorubicin/ifosfamide. They underwent CC0 surgery and HIPEC with cisplatin/doxorubicin and cisplatin/docetaxel, respectively, followed by WAP-RT. The other surviving patient initially presented with a mass in the thoracic cavity and was treated with neoadjuvant chemotherapy (VAC/IE regimen), underwent surgery, and then completed 1 year of chemotherapy.

The median PFS after first-line chemotherapy was 8.7 months (IQR: 15.5 m–4.4 m). Out of 19 patients, 13 and 11 patients received second and third lines of chemotherapy, respectively. The median PFS for patients who underwent second and third lines was 3.9 months (IQR: 9 m–2.5 m) and 2.5 months (IQR: 4.2 m–1.2 m), respectively.

Considering only the patients with abdominopelvic DSRCT (*n* = 17), 10 were submitted to CRS. In 9 of them, it was a CC0 surgery. Six patients of the ones who underwent CC0 surgery (67%) relapsed. The sites of relapse after CC0 surgery were retroperitoneum (*n* = 1), lung (*n* = 1), peritoneum (*n* = 3), and liver (*n* = 2). The median time between CC0 surgery and first relapse was 10 months (IQR: 2 m–15 m). Of the four patients who underwent complete multimodality treatment, one patient presented lung metastasis 9 months after ending of treatment and one patient developed liver and peritoneal metastasis 10 months later. The other two were alive without evidence of disease until the end of the follow-up period.

On univariate analysis, no variables (age, gender, ascites, liver metastasis, lymph node metastasis, MD Anderson stage, CRS, CC0, WAP-RT, extra-abdominal disease, tumor size, and number of lesions) were significantly correlated with OS (Supplementary Material Table 1). There was a tendency for better overall survival for patients with single lesion at presentation (mOS not reached versus 27 months for patients with multiple lesions, *p*=0.05).

## 4. Discussion

DSRCT is an extremely rare and aggressive disease. The largest populational study of DSRCT collected data from the National Cancer Database of the United States and identified 491 adult patients diagnosed with the disease between 2004 and 2014 [16]. Another study identified 192 cases between 1973 and 2007 (age 0–60 years) from the Surveillance, Epidemiology, and End Results (SEER) database [1]. In Brazil, the national database fails to detect the incidence of rare types of cancer. This is the first report evaluating the patterns of care and survival of a Brazilian cohort of patients with DSRCT and also, to the best of our knowledge, the first report of a Latin American cancer center.

From January 2007 to January 2020, we included 19 patients in the study. The minority of patients were initially managed outside our center. This series is comparable in size with other single-center experience series previously reported [3,17–20]. All patients had the diagnosis confirmed by a sarcoma pathologist. Consistent with previously published data, our results showed that DSRCT tends to occur in younger males, presenting as abdominal and/or pelvic large masses. The male-to-female ratio was 2.16:1, and the mean age at diagnosis was 26 years. Six patients (31.5%) were over 30 years old at diagnosis. Abdominopelvic DSRCT in patients older than 30 years is considered an atypical presentation, so as extra-abdominal presenting sites of disease irrespective of age, and differential diagnosis should be weighted in those cases [21]. Two patients (10.5%) had primary disease outside the abdominal cavity, one in the lung and the other in the mediastinum. Despite being unusual, extra-abdominal/pelvic DSRCTs have been documented in larger series and case reports [10,21–25]. The relative frequency of DSRCT with atypical sites of presentation is unknown [21]. It ranged between 6.6% and 24% on previous reports [3,9,10,21,25–27]. Wong et al. reported longer overall survival for patients with extra-abdominal disease compared to those with abdominopelvic DSRCT (median OS not reached vs. 15 months, respectively; *p* = 0.02) [9]. It is possible that more superficially located tumors, or located in sites that lead to early symptoms, make patients present at earlier stages of the disease, which justifies a better prognosis. In our study, we could not find a statistically significant difference between these two groups. A 22-year-old male with a 115 mm unresectable mediastinal mass, who developed a tumor-esophageal fistula, died after fatal tumoral bleeding. The other patient (34 y, male) presented a single lung mass that was initially resected, relapsing in the lung after 6 months. Thereafter, he received preoperative chemotherapy with vincristine/doxorubicin/cyclophosphamide, underwent surgery, and completed one year of adjuvant chemotherapy, staying without evidence of disease until the last follow-up, 9 years after treatment ending.

We also could confirm the aggressiveness of the disease: after a median follow-up of 95 months, the mOS of the entire population was 27 months. In the literature, the reported median survival ranges from 17 to 25 months [7]. In our cohort, 3 patients were alive without disease at the end of the follow-up period. Besides the patient with primary lung disease previously discussed, the two others presented abdominopelvic DSRCT and were retrospectively staged as 1 and 2 according to the MD Anderson proposed staging system [15]. All of them received multimodality treatment, including HIPEC and WAP-RT in those with abdominopelvic presentation. There is solid evidence that combining multiagent intensive chemotherapy and aggressive debulking surgery improves survival in patients with DSRCT, representing the standard of care, especially for patients presenting without extraperitoneal metastases [14,28,29]. Although not statistically significant, the impact of surgery could be seen in our study. Patients with abdominopelvic DSRCT stages 1 and 2 that underwent CRS presented mOS of 65 months compared to 18 months in those who not had surgery (*p*=0.36). No statistically significant difference in mOS was also found for stage 3 patients: 27 months vs. 16 months (*p*=0.4). In our cohort, one patient relapsed 2 months after CC0 surgery, which highlights the importance of assessing the patient's suitability for CRS in a multidisciplinary discussion, especially in those with higher peritoneal cancer index (PCI), since they have worse OS and the impact of surgery for them is still not clear [15,30]. It is possible that progressive disease under induction chemotherapy represents a contraindication for surgery, since chemoresistant tumors would probably quickly recur. In the report of Honoré et al., the only patient with progressive disease during neoadjuvant chemotherapy underwent a “rescue” surgery and relapsed in the peritoneum and extra-abdominal sites 6 months after surgery [31]. In the largest single-center DSRCT retrospective study, Subbiah et al. highlighted not to consider surgery for patients with progressive disease during induction chemotherapy [28].

In our study, all patients undergoing cytoreductive surgery also received chemotherapy as part of the initial treatment. Regimens combining vincristine, doxorubicin, and cyclophosphamide or ifosfamide were the most prescribed. The benefit of chemotherapy in the management of DSRCT patients has long been proved [29]. Ewing-sarcoma-based chemotherapy has indeed been the regimen of choice worldwide considering genetic and biological similarities between the two diseases [32]. But the best chemotherapy regimen is still to be defined. Kushner et al. reported 12 patients with median overall survival of 19 months with the P6 protocol (cyclophosphamide, doxorubicin, vincristine, ifosfamide, and etoposide) [29]. Those drugs had superior OS benefit also shown in other studies [9,33]. In Scheer et al.'s analyses of 3 prospective trials, VAIA protocol (ifosfamide, vincristine, doxorubicin, and actinomycin D) showed the best outcome [26].

There are scarce data on PFS after second and third lines of chemotherapy for DSRCT patients. We showed a small benefit on median PFS after those lines of therapy (3.9 and 2.5 months for second and third lines, respectively). Due to the low number of patients and the heterogeneity of treatments, it was not possible for us to conclude the real impact of second and more lines of treatment on OS, but it is reasonable to assume that new and less toxic treatments are an urgent need to prolong OS while maintaining the quality of life after the first relapse. In that way, there are emerging and promising data on targeted therapy for DSRCT. Three patients in our study received targeted therapy during treatment (bevacizumab, pazopanib, and sunitinib). The clinical evidence for use of targeted treatment for DSRCT is mainly based on case series [34]. In 2014, Frezza et al. first reported nine patients with advanced DSRCT treated with pazopanib, showing partial response in 2 patients (22%), stable disease in 5 (56%), and progressive disease in 2 (22%) [35]. Remarkably, the median PFS was 9.2 months (95% CI: 0–23.2) and OS 15.4 months (95% CI: 1.5–29.3). Pazopanib was also proven to be clinically active in heavily pretreated patients in a more recent retrospective review of MD Anderson experience [36]. In our study, a patient with liver metastasis received pazopanib in the second line, presenting progressive disease after 3 months of use. Less evidence exists for the use of sunitinib and bevacizumab, mostly based on small case series and case reports [37,38]. One ongoing trial was designed to assess the impact of the addition of irinotecan, temozolomide, and bevacizumab into conventional chemotherapy for the treatment of newly diagnosed patients [39].

The two abdominopelvic DSRCT surviving patients of our cohort also received HIPEC and WAP-RT. The role of those two treatment modalities in the management of abdominopelvic DSRCT patients is debatable. In 2010, Hayes-Jordan et al. suggested a possible longer disease-free survival for children when HIPEC with cisplatin was added to CRS [15]. Later, the same author published a study which included children and adults, showing significantly longer mOS for patients who had CR0 or CR1 and HIPEC versus CR2 and HIPEC (63 vs. 27 months) [40]. Other studies could not find a survival benefit with HIPEC [28,31,41]. Whole abdominopelvic radiotherapy (WAP-RT) was first reported as part of the multimodality treatment using the P6 protocol, with the aim of improving local control [29]. In a study including patients with primary abdominal DSRCT from 8 French centers, addition of adjuvant radiotherapy was associated with survival benefits in patients treated after cytoreductive surgery and perioperative chemotherapy [42]. In a retrospective analysis of 192 patients obtained from the SEER database, Lettieri et al. showed that radiation therapy following surgery improved patients' outcomes. Other large retrospective studies failed to detect clear survival improvement with adjuvant WAP-RT [28,41].

We suppose the low number of cases with sufficient follow-up information hindered us from assessing the correlation between pathologic and clinical findings with outcomes. Patients with a single lesion at diagnosis presented longer OS (mOS not reached versus 27 months for patients with multiple lesions, *p* = 0.05). This was not found in Angarita et al.'s study [17]. Differently, they showed extra-abdominal metastasis at the time of presentation was associated with a higher risk of mortality (HR: 3.1, *p* = 0.04). The same correlation was established by Wong et al. (mOS not reached vs. 15 months, *p* = 0.02) [9]. This finding was not associated with worse survival in other reports [8,43]. In our study, we also could not find a correlation.

The retrospective character and the small number of patients are important limitations of this study. Furthermore, only 4 patients had the diagnosis confirmed by fluorescence in situ hybridization (FISH) for EWSR1 gene rearrangement, and the identification of the EWSR1-WT1 fusion transcript by reverse transcription-polymerase chain reaction (RT-PCR) was not performed in any case. This is the real-life difficulty in diagnosing rare sarcomas in Latin American countries with limited resources. Treating patients in reference centers with access to molecular test and pathologist expertise is paramount in managing DSRCT. The establishment of cooperative groups across continents is essential to involve a larger number of patients with very rare sarcomas, such as DSRCT. The Sarcoma European-Latin American Network (SELNET consortium, granted by the European Commission within the Horizon 2020 Call) is an initiative that seeks to identify barriers in the diagnosis and treatment of rare sarcomas in Europe and Latin America [44]. More initiatives such as that need more patient advocates, government, pharmaceutical companies, and academia efforts to achieve relevant results due to rarity of this disease.

## 5. Conclusions

DSRCT is a rare aggressive soft tissue sarcoma with poor outcomes. Multidrug chemotherapy combination and surgery performed in sarcoma-specialized centers can improve survival and guide some patients to cure. Our data showed that the outcomes of patients treated in a reference cancer center in a developing country are similar to those of cancer centers in developed nations. However, the mOS of patients treated in our center with multimodal therapy is limited. In accordance with other studies, the relapse rate after surgery was high and the mPFS to second and third lines of therapy was dismal. As a very rare sarcoma, multicenter cooperation is warranted to the development of clinical trials and to improve diagnosis and treatment efficacy of DSRCT.

## Figures and Tables

**Table 1:** Patient and tumor characteristics.

Variable	Number of patients (%)
Gender	
Male	13 (68)
Female	6 (32)
Age (years), median (range)	26 (15–41)
Family history of cancer^a^	
Yes	6 (32)
No	10 (53)
Not informed	3 (15)
Symptoms at diagnosis^b^	
Pain	10 (53)
Ascites	7 (37)
Constipation	5 (27)
Weight loss	6 (32)
Dyspepsia	5 (26)
Palpable mass	2 (11)
Others (fever, cholestasis, nausea/vomits)	6 (32)
Presenting site	
Abdominopelvic cavity	17 (90)
Lung	1 (5)
Mediastinum	1 (5)
Number of tumor deposits	
Single	3 (16)
Multiple	16 (84)
Largest tumor size (cm), median (IQR)	13 (16–11)
Visceral metastasis at presentation	
Yes	11 (58)
No	8 (42)
Site of metastatic deposits (*n* = 11)	
Liver	8 (73)
Lung	3 (27)
Spleen	2 (18)
Others (bone, pancreas, breast)	4 (36)
Lymphadenopathy	
Yes	15 (79)
No	2 (10.5)
Not informed	2 (10.5)
Extra-abdominal metastasis	
Yes	6 (32)
No	13 (68)
MD Anderson staging^c^	
1	4 (21)
2	6 (32)
3	4 (21)
4	5 (26)

^a^Includes first- and second-degree relatives. ^b^All patients had symptoms at diagnosis. ^c^Based on Hayes-Jordan et al. [15].

**Table 2:** Treatment modalities.

Treatment modality	Number of patients (%)
Chemotherapy	
Neoadjuvant	9 (47)
Palliative	10 (53)
First line (*n* = 19)	
VAC/IE	7 (37)
VAC	3 (16)
VAI	3 (16)
VAC/ICE	1 (5)
AI	1 (5)
Carboplatin/ifosfamide/etoposide	1 (5)
Carboplatin/paclitaxel/bevacizumab	1 (5)
Carboplatin/paclitaxel	1 (5)
Cisplatin/etoposide	1 (5)
Second line (*n* = 13)	
IE	3 (23)
VAC	2 (15)
Pazopanib	2 (15)
High-dose ifosfamide	1 (7)
Dacarbazine/gemcitabine	1 (7)
Topotecan	1 (7)
Irinotecan	1 (7)
Sunitinib	1 (7)
FOLFOX	1 (7)
Third line (*n *= 11)	
Gemcitabine/docetaxel	3 (27)
Irinotecan	2 (18)
Topotecan/cyclophosphamide	1 (9)
Cisplatin/ifosfamide	1 (9)
VAC/IE	1 (9)
IE	1 (9)
Irinotecan/temozolomide	1 (9)
Carboplatin/paclitaxel	1 (9)
Radiation therapy	
Whole abdominal	4 (21)
Palliative	1 (5)
None	14 (74)
Surgery	
Yes	11 (58)
No	8 (42)
Hyperthermic intraperitoneal chemotherapy	
Yes	5 (26)
No	14 (74)

## Data Availability

The datasets generated during and/or analyzed during the current study are available from the corresponding author on reasonable request.

## Supplementary Materials

Supplementary Materials Supplementary Material Table 1: predictive factors for overall survival in patients with desmoplastic small round cell tumor.
