# Supplementary material for: Clinical Characteristics, Management, and Outcomes of 19 Nonpediatric Patients with Desmoplastic Small Round Cell Tumor: A Cohort of Brazilian Patients
Source: Sarcoma. 2020 Oct 28;2020:8713165. doi: 10.1155/2020/8713165 (PMC7643375; doi:10.1155/2020/8713165)
Supplement: Supplementary Materials — Supplementary Material Table 1: predictive factors for overall survival in patients with desmoplastic small round cell tumor. [file 8713165.f1.docx]

**Supplementary Material Table 1 – Predictive factors for overall survival in patients with desmoplastic round cell tumor**

| **Variable** | **HR** | **95% CI** | **p-value** |
| --- | --- | --- | --- |
| Age < 20 years^a^ | 2.3 | 0.7 – 7.7 | 0.2 |
| Male gender | 0.4 | 0.1 – 1.4 | 0.2 |
| Single lesion | 0.2 | 0.02 – 1.3 | 0.09 |
| Tumor mass < 13 cm^b^ | 1.8 | 0.6 – 6.4 | 0.4 |
| Liver metastasis | 2.7 | 0.5 – 13.2 | 0.2 |
| Ascites at diagnosis | 2.1 | 0.6 – 6.7 | 0.2 |
| Extra abdominal disease | 1.0 | 0.3 – 2.9 | 0.9 |
| Lymph node impairment | 3.4 | 0.4 – 26.7 | 0.2 |
| MD Anderson staging system  Stage 1  Stage 2  Stage 3  Stage 4 | Reference  2.2  2.8  4.1 | -  0.3 – 13.3  0.5 – 15.8  0.7 – 24.3 | 0.5  0.4  0.2  0.1 |
| Cytoreductive surgery | 0.5 | 0.2 – 1.4 | 0.2 |
| Complete cytoreductive surgery | 1.0 | 0.1 – 9.0 | 0.9 |
| Adjuvant radiotherapy | 0.6 | 0.1 – 3.2 | 0.6 |

**﻿**

Abbreviations: 95% CI, 95% confidence interval; CI, confidence interval; HR, hazard ratio.

^a^Cut off is based on the value that maximizes the log-rank test.

^b^Cut off is based on median.
